# Exploring Self-Efficacy in Australian General Practitioners Managing Patient Obesity: A Qualitative Survey Study

**DOI:** 10.1155/2016/8212837

**Published:** 2016-05-04

**Authors:** Freya Ashman, Elizabeth Sturgiss, Emily Haesler

**Affiliations:** Australian National University Medical School, Academic Unit of General Practice, P.O. Box 11, Woden, ACT 2606, Australia

## Abstract

*Background.* Obesity is a leading cause of morbidity and mortality in the Australian community, and general practitioners (GPs) are commonly approached by patients for assistance in losing weight. Previous studies have shown that GPs have low self-efficacy and low outcome expectation when it comes to managing overweight and obese patients, which affects their willingness to initiate and continue with weight counselling. This qualitative survey study aimed to explore the factors influencing confidence and behaviour in obesity management in GPs.* Method.* Twelve GPs recruited to deliver a pilot of an obesity management program participated in semistructured interviews, and interpretive analysis underpinned by social cognitive theory was performed on the transcripts.* Results.* Analysis identified five main themes: (1) perceived knowledge and skills, (2) structure to management approach, (3) the GP-patient relationship, (4) acknowledged barriers to weight loss and lifestyle change, and (5) prior experience and outcome expectation.* Conclusions.* GPs are likely to welcome tools which provide a more structured approach to obesity management. Shifting away from weight and BMI as sole yardsticks for success or failure and emphasising positive lifestyle changes for their own sake may improve GP self-efficacy and allow for a more authentic GP-patient interaction.

## 1. Introduction

The Global Burden of Disease study in 2010 estimated overweight and obesity as secondary only to dietary factors (such as diets high in fat and salt and low in vegetables) in terms of risk for burden of disease in Australasia, placing it above even cigarette smoking [1]. The proportion of Australian adults classified as overweight or obese by body mass index (BMI) is increasing and currently includes nearly two-thirds of the population [1]. Similar rates of overweight and obesity to the national prevalence have been seen in patients attending general practitioners (GPs), with Australian data suggesting that over 3 million obese adults attended GPs (for any reason) over the period of 2006–2008 [2].

Patients do believe that the GP has a role to play in weight management [3]. Various guidelines exist to inform GP management of overweight and obesity [4, 5], though there is a lack of evidence in the literature for any lifestyle intervention achieving significant, sustained weight loss [6–8]. This has contributed to an increasing amount of discussion in the literature of newer obesity paradigms such as the Health at Every Size movement, which aims to reduce stigma and dehumanisation of overweight and obese people and to move from a weight-focused approach to a broader health-focused one [9, 10].

A large amount of literature exists around GP attitudes to management of overweight and obesity in adult patients. Barriers to effective care in this area commonly identified by GPs include time constraints, lack of adequate training, frustration at previous outcomes, and poor patient compliance or motivation [11–15]. A study of rural Australian GPs identified the relative lack of health resources available in rural areas as a barrier [16]. In addition, an interview study of GPs in the UK identified as barriers the conflict between GP and patient opinions about who had the primary responsibility for managing the patient's obesity and the potential strain the GP's efforts placed on the GP-patient relationship [17].

*Self-efficacy* refers to the perception that people have of their ability to perform the actions necessary to achieve a desired outcome. Multiple mechanisms by which self-efficacy beliefs can be influenced have been identified. These include direct mastery experiences (performing the action oneself), vicarious experiences (observing other people performing it), imagined experiences (e.g., visualisation exercises), and verbal or social persuasion (being told that the action is possible) [18, 19].

Studies have consistently agreed that GPs have low self-efficacy and low expectation of effective treatment in the area of weight management [20–22]. Insufficient confidence has been quoted as a barrier both to delivering weight loss counselling and to raising the issue of weight with patients initially [23–25]. Studies have shown associations between both high self-efficacy and high outcome expectation in this area and the amount of counselling performed by the doctor [26, 27]. A systematic review in 2011 found that health professionals felt underprepared and lacking sufficient knowledge to deal with obesity and that they considered current approaches to treatment to be ineffective [28]. A 2000 survey of Australian GPs found that they considered themselves to be well prepared to treat overweight patients, although they believed that they have limited efficacy in weight management and found it professionally unrewarding [11].

The majority of the aforementioned studies take the form of surveys. A number of studies have used focus groups [13, 29] or interviews [14, 17, 22, 30] to look in more depth at the experiences and attitudes of GPs in this area of health care. However, none were found to have done so in an Australian context.

“The Change Program” [31] is a GP-delivered weight management program to assist in the management of adults who are overweight and obese in primary care. The program is based on evidence-based Australian guidelines for obesity management. It acknowledges that for some patients the involvement of allied health practitioners in this care may not be possible for a variety of reasons (e.g., time, cost, and location) and also aims to leverage the unique GP-patient dynamic to effect behaviour change.

This study aimed to explore the factors influencing confidence and behaviour in obesity management in GPs recruited for a pilot study of The Change Program. This investigation used a social cognitive theory perspective and specifically explored GP self-efficacy in overweight and obesity management.

## 2. Methods

This was a qualitative survey study using semistructured interviews, drawing on the theoretical framework of social cognitive theory (SCT). This is a theoretical framework, first described by the social psychologist Bandura in 1977 [32], which has since been commonly employed in the field of health promotion [18]. The theory outlines a number of determinants of behaviour that include self-efficacy, outcome expectation, and perceived barriers and facilitators.

Ethics approval was gained through the ANU Human Research Ethics Committee.

Twelve GPs recruited via a snowball sampling strategy to deliver a pilot of an obesity management program (“The Change Program”) participated in semistructured interviews before commencing the pilot. Of the 12 participating GPs (four male and eight female), ages ranged between 31 and 60, and years working in general practice ranged between 4 and 30. The GPs were from five different practices, with two of the GPs practising in a rural New South Wales location and the other ten practising within the urban environment of the Australian Capital Territory.

Interviews were face-to-face and were conducted in March and April of 2015. All interviews were conducted by the lead researcher (FA), who is herself a clinically active general practitioner, and informed consent was gained from participants prior to interview. A self-efficacy questionnaire adapted from previously validated tools [33–35] was administered at the start of the interviews (see the Appendix). It comprised a 4-point Likert scale with items loosely arranged under the framework of the “5As” (assess, advise, agree, assist, and arrange), in line with the current guidelines for obesity management in general practice [5]. The GPs were asked about their current management of obesity and read two short clinical vignettes to prompt reflection on their own practice and then were asked to discuss those items on the questionnaire where they had indicated they were “less confident” or “not at all confident.”

Interviews were recorded digitally and transcribed verbatim by a professional transcription service. Deidentified data were transferred into NVivo 10 for data organisation and coding. Data were analysed independently by two researchers (FA and ES, both general practitioners) and coded using social cognitive theory as a guiding framework. Findings were discussed by the two researchers in order to reach consensus, with any areas of discrepancy discussed with a third researcher.

## 3. Results

Five main themes emerged from the data in regard to factors influencing GP self-efficacy in the management of obesity.

### 3.1. Skills and Knowledge

Several of the GPs felt that there were specific gaps in their knowledge or skillset which lowered their confidence.

*I think for people…who are considerably overweight I don't understand enough about the effect of physical exercise on their bodies to be confident of giving them a good exercise program. (S07)*

Other examples included lack of awareness of the specific caloric value of foods, inexperience in providing advice on diet in obesity in combination with other diet-specific management problems (e.g., high cholesterol or iron deficiency), and not knowing target heart rates for exercise.

The majority of the GPs stated that they often referred patients to allied health specialists such as dieticians or exercise physiologists or recommended commercial weight loss programs.

*I don't feel confident to really get into the nitty gritty of… patients' questions about this diet, this food and that food, and I think, oh, I'll leave that to somebody else to do. (S04)*

### 3.2. Structure to Approach and Follow-Up

Many GPs felt that their approach to managing obesity was disjointed or opportunistic. The GPs stated that this lack of structure made them less confident in their obesity management, and they expressed an interest in The Change Program as something that might address this particular issue.

*It comes down to follow up, I think, and feeling confident that I have a system in place that I'm going to see them and act on. I feel like a lot of my obesity management has been very opportunistic with not a lot of protocol or system around it. (S09)*

They spoke confidently about initiating the conversation about weight and giving counselling to the patient at that time but admitted they often did not make concrete plans to follow up the patient's progress.

*I'd say it's just really ad-hoc. So, we'll talk about it here or there, but not ‘we said we'd do this, did we?' kind of thing. Sometimes they won't come back for six months. (S05)*

### 3.3. The GP-Patient Relationship

The unique therapeutic and often long-term relationship between a GP and their patient was mentioned as impacting GP behaviour and confidence. GPs can have comprehensive knowledge of the patient's personal situation, medical comorbidities, and possible barriers to lifestyle change. This was seen by most of GPs in this study both as a time saver (with less history taking and teasing out of individual challenges required) and as facilitating the GP's ability to counsel and empower patients to change. Some GPs described a good-quality and trust-based therapeutic relationship between themselves and the patient as improving their personal confidence in their counselling ability by acting as a facilitator of open disclosure and honesty from the patient, allowing for a more accurate gauge of a patient's state of change and current lifestyle and perceived by the GP as providing “permission to engage.”

*[Having an established relationship] makes it easier because you know what works and…what makes this patient tick…With a patient that you've seen over many years you know where they stand…You can be more frank with them I suppose, and you know their life situation. (S11)*

Conversely, some GPs spoke about what could happen when their relationship with the patient actually hindered their management attempts.

*You then stop doing it with certain people if you're finding that…they put up the barriers and that you've done the same discussion over and over again. It might be hard to readdress that with the same person ‘cause you feel like you're battering them a bit. (S06)*

A few of the GPs reflected that the possibility of patients feeling guilty or reprimanded if they failed to lose weight might contribute to patient failure to attend follow-up or negatively affect the GP-patient relationship.

*I think if patients haven't been successful they generally don't turn up on those follow up appointments as well. And I know of some patients who… actually put on weight instead of losing weight…that patient will now come and see me ‘cause they don't want to see [their usual GP] ‘cause they feel like they're going to be in trouble. (S03) *

### 3.4. Acknowledging Barriers to Weight Loss

Most GPs expressed a strong awareness of multiple barriers to weight loss that their patients might experience, which tended to negatively affect the GPs' confidence.

*I look at the social difficulties for them to lose weight, whether they're the person who does the cooking or the shopping in the family, who else is at home, who else are they having to cater for…what sort of work she's doing, whether it's sedentary work or not… Whether she actually has any free time for herself or whether her whole life revolves around work and shopping and rushing the children around. (S07)*

They showed awareness of background factors contributing to obesity, such as the “obesogenic environment” and an increasingly sedentary society but also discussed many different social and lifestyle factors that might obstruct change for their patients. Commonly quoted examples were the impact of a patient's family context, the distribution of roles such as shopping and cooking, the easy availability of high-calorie food, and time pressure around work and home duties.

Another acknowledged barrier, which some GPs reported as negatively affecting their confidence and decreasing their expectation of weight loss outcomes, was their awareness of the lack of evidence for any lifestyle intervention to cause significant sustained weight loss. Several GPs also mentioned “biological set points” and the tendency of the body to regain weight in the long term even if some weight loss was achieved, and they also showed awareness of this lack of evidence in the literature.

*I don't want to be falsely saying…‘I really believe if you do this this would be effective'… I just think if people have put on weight often their body's fighting to get back up to that weight… and I know some people lose weight and they do keep it off with a lot of effort, but I think the majority of people put it back on… So I don't feel confident empowering people. (S03)*

Some of the GPs in this study discussed making attempts to readjust patient expectations if, for example, a patient expressed a desire to lose an amount of weight that the GP considered particularly unrealistic.

### 3.5. Prior Experience and Outcome Expectation

Almost all GPs reported that their confidence was negatively affected by their failure to successfully achieve weight loss in most of their patients with obesity and their subsequent low expectation of being able to achieve this in the future.

*I've been treating patients who are overweight or obese for years, and I wouldn't say that my success rate is particularly high…in seeing my patients lose weight, so that's why I don't feel particularly confident that I'm good at it…I've made suggestions that I think would be effective, but in so many times… we don't actually get very far. (S07)*

In some cases, the GPs* had* experienced success in assisting their patients to achieve weight loss, which was accompanied by increased confidence. However, these instances were heavily outweighed in the data by GPs speaking negatively about failure of their patients to achieve weight loss outcomes and expressing feelings of frustration and low confidence in managing their patients' obesity as a result.

Some GPs mentioned using their own personal experiences in behaviour change and weight loss in order to motivate patients.

*I've heard of the 5:2 diet, and I sort of practice a little bit of it. I share my own experience as well … invite them to try what I'm trying. (S11)*

In addition, a few GPs brought up alternative (i.e., not weight loss per se) outcomes and endpoints, such as considering patients' blood pressure or fasting blood results, aiming for a lack of weight* gain*, or simply having raised the issue and provided patient education as a “good result.”

## 4. Discussion

Based on the results from this study, GPs' self-efficacy for obesity management is affected by their previous experiences and practice, their perception of the low level of evidence for interventions achieving successful long-term weight loss, and their perceived level of knowledge and skills. These results from the Australian context provide support for findings from existing international research.

The perceived efficacy of the GPs' counselling skills was affected by a series of barriers and facilitators. These included the presence or absence of a structured approach to weight management in practice and awareness of the larger context such as the obesogenic society but also the therapeutic relationship that exists between GP and patient. General practitioners perceived this relationship as being important for the level of trust the patient placed in them, the doctors, to act in the patient's best interest. They also identified that relationship as influencing how much they know about the patient's own self-efficacy and personal life circumstances (which could influence the patient's lifestyle change attempts).

The GPs were pragmatic and sometimes pessimistic about the limits of their own influence in the context of everyday practice. They showed awareness that their own knowledge base and their skills, for example, in motivational interviewing, were the factors over which they had the most control. This has highlighted an issue in considering GP management of obesity from a social cognitive theory perspective: in essence, it is a single-body psychological framework being applied to a two-body problem. The therapeutic relationship between those two bodies further muddies the waters by potentially acting as a barrier or facilitator (or both) in its own right. The possible strain that efforts to manage obesity could place on the GP-patient relationship was also identified in a 2005 study by Epstein and Ogden of UK GPs [17].

In a sense, too, patient self-efficacy appeared to influence GP self-efficacy. In many cases the* GP* was less confident as a result of barriers to behaviour change their* patient* faced. The interviewed GPs actually reported strategies in keeping with social cognitive theory, which they employed in order to increase patient self-efficacy, for example, modelling the advised behaviours (“this worked for me!”), motivational interviewing as a form of delivering imagined experience, and discussing with the patients the recognised barriers to mastery experiences.

As has been pointed out already, the low expectation of positive weight loss outcomes from the GP's actions is supported by the evidence in the literature [7, 8], in which few lifestyle interventions have been shown to produce significant, sustained weight loss. Some of the GPs were aware of this, and this lack of evidence can be considered as impacting on self-efficacy both as outcome expectation and as a form of verbal persuasion: being told by an external source that “this isn't possible.”

A lot of the frustration in the GPs arose from this depressing cycle of not being effective and therefore not feeling confident, giving rise to an unusual situation of often not genuinely* believing* that the management advice that they were giving patients would produce the desired outcome, which can be contrasted with the treatment advice given for many medical conditions. This is consistent with findings from a 2014 study in New Zealand of GP perceptions around weight management, which found that GPs felt disempowered by the lack of efficacy of available resources and interventions [30], and a 2007 study of primary care clinicians in the US, in which the interviewees expressed frustration over lack of effectiveness in this area [22].

This frustration sets up a conflict for the GP between their desire to provide encouragement and motivation and the fact that they cannot in full honesty provide high weight loss outcome expectations to the patient. As mentioned, some GPs made an attempt to compromise here by trying to adjust patient expectations around implausible goals for weight loss. A 2011 interview study of GPs and nurses in Scotland [14] similarly found that some clinicians encouraged change for its own sake rather than pushing for unrealistic goals, and the authors identified that if modest weight loss or even just preventing weight gain is seen as success, then this may ameliorate the GPs' sense of frustration.

One limitation of this study is that no participant checking was conducted in order to verify the accuracy of our analysis of participant comments. In addition, this was a qualitative survey study looking at a small group of recruited GPs from a limited geographic region and is not expected to be representative of the whole breadth of Australian general practice experience. The nature of recruitment meant that the GPs involved were likely to have a particular interest in improving their management in the area of overweight and obesity, compared to the wider GP population. Given that recruitment was via convenience sampling (part of a wider study), data saturation was not confidently reached. However, few new themes emerged in the final interviews.

Finally, as both researchers involved in interview and analysis were GPs themselves, they had a shared language and context with the participants, which may have influenced both the participants' responses and the researchers' interpretations. This is likely to have increased the depth of responses and the integrity of the thematic analysis and can therefore be considered a strength of the study.

## 5. Conclusions

When it comes to improving GP confidence around management of obesity, this study suggests a small role for addressing areas of knowledge that GPs might want to improve in order to feel more confident helping patients themselves instead of referring directly to allied health practitioners. In addition, given that the participants involved were a presumably motivated and interested subgroup of GPs, these perceived knowledge gaps may be even more of an issue in the wider GP population.

The findings also suggest that GPs are likely to welcome any tools or support when it comes to having a structured approach to the management of overweight and obesity in practice. To this end, the same GPs will be interviewed after the six-month pilot study of “The Change Program” and will be directly asked about whether the structure inherent in the program had an effect on their confidence in this area.

Finally, given the lack of clinical evidence for even our “best” strategies to assist patient weight loss, the question does need to be posed: should we be changing the goalposts when it comes to obesity management? For GPs to break that cycle of feeling like they and their patients are trying to climb an unassailable slope, without ever seeing “success,” options would be as follows: focusing more on medical endpoints such as fasting glucose or blood pressure, aiming for a* maintenance* of current weight (over- or otherwise), or aiming for lifestyle changes such as increased physical activity which have recognised health benefits independent of weight. This area of possible future exploration is in line with the Health at Every Size paradigm [9, 10].

Shifting the frame away from weight loss per se and towards these other goals might therefore allow for a more authentic GP-patient interaction and increase the self-efficacy of both GP and patient for healthy lifestyle changes.

## Appendix

### Obesity Management Survey

This survey aims to look at your attitudes to managing overweight and obese people in your everyday practice and how confident you are at various aspects of management.

Please rate your agreement with the following statements:

I feel qualified to treat overweight/obese patients
  1 (*Strongly disagree*)
  2 (*Disagree*)
  3 (*Agree*)
  4 (*Strongly agree*)
People with overweight/obesity have difficulties adjusting their lifestyle
  1 (*Strongly disagree*)
  2 (*Disagree*)
  3 (*Agree*)
  4 (*Strongly agree*)
As a GP, I have little influence on the lifestyle of people with overweight/obesity
  1 (*Strongly disagree*)
  2 (*Disagree*)
  3 (*Agree*)
  4 (*Strongly agree*)
I know enough about nutrition to give education for treatment of overweight/obesity
  1 (*Strongly disagree*)
  2 (*Disagree*)
  3 (*Agree*)
  4 (*Strongly agree*)
I know enough about physical activity to give education to patients for treatment of overweight/obesity
  1 (*Strongly disagree*)
  2 (*Disagree*)
  3 (*Agree*)
  4 (*Strongly agree*)
I am confident that my counselling makes a difference for my overweight/obese patients
  1 (*Strongly disagree*)
  2 (*Disagree*)
  3 (*Agree*)
  4 (*Strongly agree*)
In general, I am confident in my ability to treat patients who are overweight/obese
  1 (*Strongly disagree*)
  2 (*Disagree*)
  3 (*Agree*)
  4 (*Strongly agree*)

At this point, how confident are you in your ability to perform the following tasks?

Assess a patient's readiness to change their behaviour and lifestyle
  1 (*Not at all confident*)
  2 (*A little confident*)
  3 (*Confident*)
  4 (*Very confident*)
Screen for comorbidities commonly associated with overweight/obesity
  1 (*Not at all confident*)
  2 (*A little confident*)
  3 (*Confident*)
  4 (*Very confident*)
Discuss the general effects of obesity on present and future health
  1 (*Not at all confident*)
  2 (*A little confident*)
  3 (*Confident*)
  4 (*Very confident*)
Personalise the discussion of risk to each patient
  1 (*Not at all confident*)
  2 (*A little confident*)
  3 (*Confident*)
  4 (*Very confident*)
Counsel a patient on the benefits of a healthy lifestyle
  1 (*Not at all confident*)
  2 (*A little confident*)
  3 (*Confident*)
  4 (*Very confident*)
Counsel a patient on nutrition in the context of treating overweight/obesity
  1 (*Not at all confident*)
  2 (*A little confident*)
  3 (*Confident*)
  4 (*Very confident*)
Counsel a patient on physical activity in the context of treating overweight/obesity
  1 (*Not at all confident*)
  2 (*A little confident*)
  3 (*Confident*)
  4 (*Very confident*)
Identify obstacles and barriers to change in a patient's life
  1 (*Not at all confident*)
  2 (*A little confident*)
  3 (*Confident*)
  4 (*Very confident*)
Address and challenge obstacles and barriers to change in a patient's life
  1 (*Not at all confident*)
  2 (*A little confident*)
  3 (*Confident*)
  4 (*Very confident*)
Use motivational interviewing to help change behaviour
  1 (*Not at all confident*)
  2 (*A little confident*)
  3 (*Confident*)
  4 (*Very confident*)
Collaborate with a patient to set realistic goals
  1 (*Not at all confident*)
  2 (*A little confident*)
  3 (*Confident*)
  4 (*Very confident*)
Tailor an overweight/obesity management plan to an individual patient
  1 (*Not at all confident*)
  2 (*A little confident*)
  3 (*Confident*)
  4 (*Very confident*)
Include a patient in the decision-making and management process
  1 (*Not at all confident*)
  2 (*A little confident*)
  3 (*Confident*)
  4 (*Very confident*)
Empower a patient to change their behaviour and lifestyle
  1 (*Not at all confident*)
  2 (*A little confident*)
  3 (*Confident*)
  4 (*Very confident*)
Use the NHMRC (National Health and Medical Research Council) guidelines for management of overweight/obesity in your daily practice
  1 (*Not at all confident*)
  2 (*A little confident*)
  3 (*Confident*)
  4 (*Very confident*)
Create a therapeutic environment where a patient will feel understood and supported
  1 (*Not at all confident*)
  2 (*A little confident*)
  3 (*Confident*)
  4 (*Very confident*)

End of survey.
